# Pro- and Antioxidative Effect of α-Tocopherol on Edible Oils, Triglycerides and Fatty Acids

**DOI:** 10.1007/s11746-013-2227-y

**Published:** 2013-03-15

**Authors:** Maria Jerzykiewicz, Irmina Ćwieląg-Piasecka, Adam Jezierski

**Affiliations:** 1 Faculty of Chemistry, Wroclaw University, F. Joliot-Curie 14 St., 50-383 Wroclaw, Poland; 2 Institute of Soil Science and Environmental Protection, Wroclaw University of Life and Environmental Sciences, Grunwaldzka 53 St., 50-357 Wroclaw, Poland

**Keywords:** Oils, Fatty acids, α-Tocopherol, EPR

## Abstract

Using advanced electron paramagnetic resonance techniques (EPR), oxidation of crude vegetable oils and their components (fatty acids and triglycerides) by radicals generated from hydrogen peroxide was investigated. The correlation rotational times were determined allowing us to characterize radicals formed during edible oils oxidation. Additionally ^1^H- and ^14^N-hyperfine coupling constants differentiate the fatty acids dependently on their unsaturation. The acids with a higher number of unsaturated bonds exhibit higher A_N_ values of PBN/·lipid adduct. The waste oil with high free fatty acids content underwent the oxidation reaction more efficiently, however due to saturation and the high content of the fatty acids the carbon-centered radicals formed (upon hydrogen peroxide radicals) and their PBN (*N*-*tert*-butyl-α-phenylnitrone) adducts were less stable. The antioxidant effect was dependent on the amount of α-tocopherol added. In small amounts of up to 0.35 mg/1 g of fatty acid or triglyceride, it inhibited the creation of PBN/·lipid adducts while with higher amounts it intensified adduct formation. The α-tocopherol (AT) addition influence was also studied as spin scavenging dependence and indicated that any addition of the antioxidant in the investigated samples led to free radical scavenging and the effect increased with the increase in AT content.

## Introduction

The principal features of oils—their stability and quality are dominated by their lipid characteristics. The oxidation of the lipids, although studied for many years, is still not a fully understood process [1–5]. The lipid structure, especially a content of the saturated, mono- or poly- unsaturated triglycerides, is the main indicator of oils oxidative stability and has already been widely investigated [2–6]. An additional factor which influences oxidation processes of the oils is the antioxidants content. Antioxidants occur naturally in oils and belong mainly to the tocopherols group. α-tocopherol is considered to be the predominant antioxidant in olive and sunflower oils [8–10] while γ-tocopherol prevails in rapeseed oil [8–10].

**Scheme 1:**
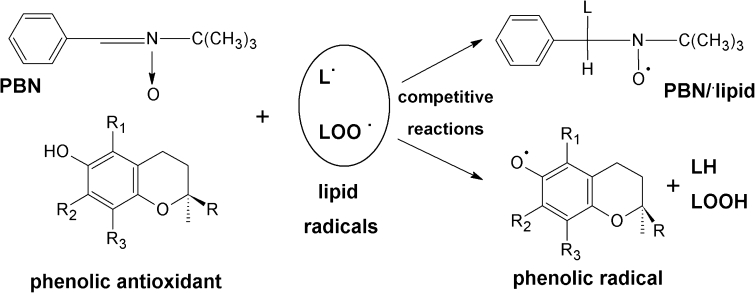
Competitive reactions of lipids radicals with a PBN spin trap and phenolic antioxidant

The tocopherols have been studied as interferents of chain-breaking initiation or donors of hydrogen atoms to the lipid peroxyl or alkoxy radicals. The pathway of antioxidative processes in oils depends on the type and concentration of the tocopherol homologue and the oils saturation [11]. The antioxidation process is based on the scavenging of radicals of lipid origin by the tocopherols, in particular by α-tocopherol (AT), leading, in general, to transformation of these antioxidants to new ·AT radicals. These radicals may further initiate oxidation, implying the well-known dilemma of prooxidant properties of tocopherols [12, 14]. The process is especially intense when the concentration of α-tocopherol is high, however, other antioxidants present in the system can restrain this prooxidant activity by regeneration of the ·AT radicals to α-tocopherol [11]. The transformations occurring during lipids oxidation (with antioxidant or prooxidant routes) are based on the formation of the free radicals [11, 14]. These radicals, provided that they have stable structure like α-tocopherol radicals, are suitable for direct EPR studies [7, 15]. Notwithstanding, as most of the radicals created upon oxidation are unstable, their investigation is possible only by using the EPR spin trapping technique [16]. The most popular spin trap in the studies of oils oxidation is PBN, due to its good solubility in the lipids, stability and low toxicity [1]. Apart from the trapping of the temporary radicals, the PBN also acts as their scavenger, so when PBN and antioxidants are present in the solution, a kinetic competition; presented in Scheme 1; occurs [17]. The effect of antioxidant-lipid-PBN interaction depends on the type of oil and antioxidant [8].

The EPR spectra of PBN radical adducts (in contrast, for example, to DMPO) are dominated by the hyperfine splitting from the nitroxide nitrogen, similarly to the EPR spectra of standard spin labels [18, 19]. The spectra of PBN adducts may differ in the hyperfine splitting constants and depend on the solvent polarity (especially on the Et (30) parameter) [19, 20]. This effect has not been studied yet for larger-sized radicals such as lipid radicals.

Additionally, the change of line shape characteristic of the low-, intermediate- or high-field signals of ^14^N-hyperfine splitting triplet allows us to calculate the correlation time, was usually examined to probe the motional dynamics of the paramagnetic species, particularly in spin-labeling studies. Estimation of the correlation times for oils, fatty acids and triglycerides can be also helpful in the identification of the radical trapped and labeled by PBN.

In this paper we present the results of EPR spectral studies on the oxidation processes of various rapeseed and waste soybean oils and their simpler, expected components: linolenic, linoleic and oleic acid, glyceryl trilinoleate, glyceryl trioleate and α-tocopherol. The role of α-tocopherol in oxidation, together with the effect of its concentration, is deemed to be especially important. The structural properties of the radicals formed upon oxidation were analyzed, also taking into account the correlation times of the PBN adducts with the radicals generated in the oils and in their probable components as references.

## Materials and Methods

### Reagents

Three different rapeseed oils were used: two of them (O1 and O3) were cold-pressed and one, the O2, was refined. The O2 sample had the lowest free fatty acid (FFA) content (0.04 %), while O1 and O2 being cold-pressed oils had a higher FFA content (0.3 and 0.4 %, respectively). These commercial oils although having similar parameters (saponification number 185–200, peroxide number 0–5) came from different local Polish producers. The producers did not use any additives such as antioxidants. As a comparative material, O1 oil was also used after 2 h of deep-frying- O1 W. A mixture of waste cooking oils from different restaurants was also investigated (MW). As the source of waste oil was diversified the mixture MW contain different oils (rapeseed, soybean, palm) and some different antioxidants could be used by producers as additives. Water contamination in this sample was low (about 1 %) and was additionally distilled off before the analyses. The acid number of MW was 5.2. This sample composed of: triglycerides (94.5 %), diglycerides (2.7 %) and free fatty acids (C-18 unsaturated mostly, 1.76 %). Linolenic acid (LIN3), linoleic acid (LIN2) oleic acid (OLA), glyceryl trilinoleate (trilinolein, TRILIN), glyceryl trioleate (TRIOLA), α-tocopherol (AT), *N*-*tert*-butyl-α-phenylnitrone (PBN) and galvinoxyl radicals were purchased from Sigma–Aldrich. Hydrogen peroxide and solvents: ethyl alcohol (AL), acetone (AC) and ethyl acetate (EA) were purchased from POCH S.A. (Polish Chemical Reagents).

### Spin Trapping Assay

A 0.5-mL sample of 0.067 mol/L PBN (*N*-*tert*-butyl-α-phenylnitrone) spin trap solution in three different solvents: ethyl alcohol (AL), acetone (AC) and ethyl acetate (EA), was added to 250 mg of oils, triglycerides or fatty acids to obtain homogeneous solutions. Afterwards, each resulting solution was mixed with 0.125 mL of 0.9 mol/L hydrogen peroxide solution to initiate the oxidation processes. Blank samples of the solvents were also studied. In order to study the impact of α-tocopherol (AT) concentration on the oxidative properties of the oils, triglycerides and fatty acids, the different quantities of AT, from 0.16 to 40 mg/g of the samples, were employed. In each case the total mass of the sample and AT was the same. In the case of smaller amounts of α-tocopherol added, appropriate solutions of this antioxidant were prepared and its aliquots added to the mixture being examined. The samples with α-tocopherol addition were named by AT e.g. when α-tocopherol was mixed with LIN the resulting sample name was LIN-AT. The samples were measured 1 h after addition of H_2_O_2_ to the homogeneous mixtures described earlier. The reaction mixtures were next sampled and EPR spectra were measured every day during the first week, then every few days and finally every week until the complete disappearance of the radical signal. The spin trapping experiments were repeated at least five times for each sample.

### Free Radical Scavenging Assay

**Scheme 2:**
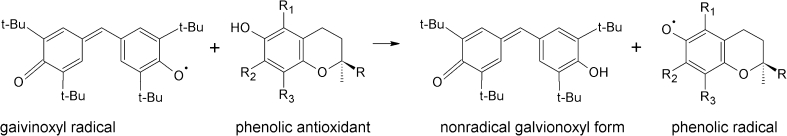
Reaction of galvinoxyl radical with a phenolic (tocopherol like) antioxidant

The free radical scavenging was performed with the galvinoxyl radical (Sigma–Aldrich) as a quenched radical according to the method that had previously been developed and described by us [21]. The studied reaction between radical and antioxidant is presented in Scheme 2. The sample of oil or fatty acid (19 mg) was diluted with 200 μL of hexane and was used as a galvinoxyl radical scavenger. The samples with addition of α-tocopherol were also prepared: 20 μL of α-tocopherol in hexane (5.1 mmol/L) and 180 μL of hexane were added to 19 mg of the samples with LIN3 and O2 giving LIN3-AT and O2-AT, respectively. Additionally, an AT sample consisting of 20 μL of α-tocopherol in hexane (5.1 mmol/L) and 180 μL of hexane, was investigated. Just before the measurements, to these prepared mixtures, 200 μL of galvinoxyl in hexane (1.1 mmol/L) was added. The EPR signal was measured and recorded immediately and every few minutes till the final disappearance of the galvinoxyl signal. As a reference, the sample containing 200 μL of hexane and 200 μL of the galvinoxyl solution was used.

### EPR Measurements

The EPR measurements were performed for the samples in glass capillaries (0.8 mm i.d.) which were kept in standard EPR quartz tubes, with at least five replications. The EPR spectra were obtained using a Bruker Elexsys E500 spectrometer equipped with an NMR teslameter (ER 036TM) and frequency counter (E 41 FC) at the X-band at room temperature. Microwave power of 20 mW, modulation amplitude of 1 G and double resonance cavities (TD104) were used. Smaller amplitudes and powers were also tested, but the above-mentioned parameters were found to be the most suitable for our samples and instrumentation. The reference samples for scavenging experiments were placed in the first cavity of the double resonator, while the analyzed sample was placed in the second one. The EPR spectra of the samples in both cavities were measured over the same period of time. Measurements were repeated at least five times. The spectra obtained in each replication were double integrated computationally. Then the arithmetic averages of calculated intensities was used to compare the data with reference samples intensities.

The first spectra from spin trapping assay were observed about 1 h after addition of H_2_O_2_ to the homogeneous mixtures. These spectra obtained for the PBN spin trapping procedure were additionally analyzed to calculate the rotational correlation time τ(s)[22, 23] according to the equation:

$$ \tau_{R} = 6.51 \times 10^{ - 10} [({\text{h}}_{0} /{\text{h}}_{ + 1} )^{1/2} + ({\text{h}}_{0} /{\text{h}}_{ - 1} )^{1/2} ]\Updelta {\text{H}}_{0} $$

where, ΔH is the middle line width of the resulting triplet (in Gauss) and h_+1,_ h_-1_ are the heights of the low and high field lines, respectively.

As the experiments were repeated five times, the rotational times results presented in the text are the arithmetic average of calculations done for five measurements.

The computer simulations of the experimental spectra were performed using the WinEPR SimFonia program (1.26 version) developed by Bruker. For calculation of the concentration (double integration of the EPR signal) and rotational correlation time (line width and height) a Bruker WinEPR 2.22 program was applied.

## Results and Discussion

### Free Radicals Scavenging Properties of Oils and Fatty Acids

**Fig. 1:**
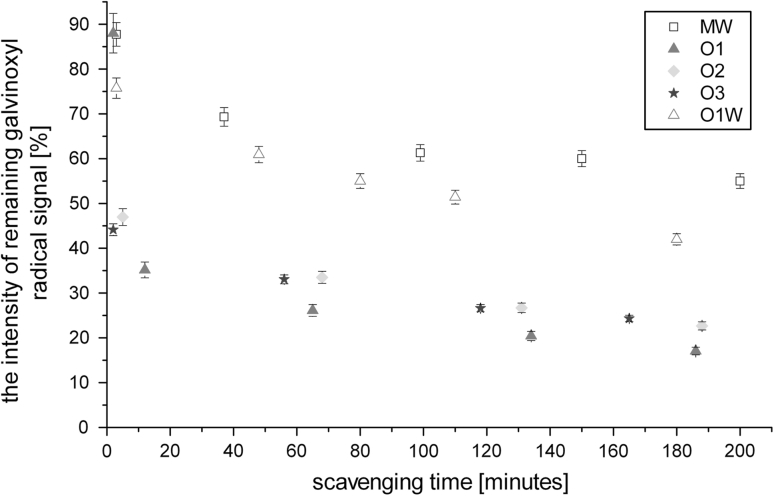
Dependence of galvinoxyl radical scavenging (measured by the intensity of the remaining galvinoxyl radical signal) for different oil samples versus time)

All of the investigated rapeseed oil samples exhibited quite similar scavenging properties towards the galvinoxyl free radical. More significant difference was observed only for waste oil (W) whose quenching ability was dramatically decreased comparing with the samples of fresh rapeseed oil, never exceeding more than 50 % of galvinoxyl radical scavenging (Fig. 1). This ineffectiveness of the waste oil in radical scavenging can be easily explained as a result of the antioxidant exhaustion by previous oil cooking and frying. What is more, the edible oils destined for extended periods of cooking are mixtures of saturated oils, hence they are more resistant to oxidation and in consequence quenching of galvinoxyl radical.

**Fig. 2:**
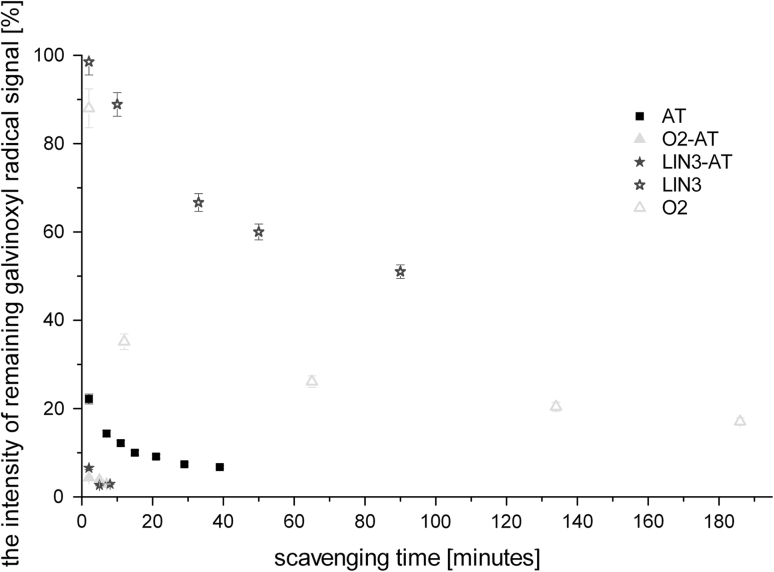
Dependence of the galvinoxyl radical scavenging ability (measured by the intensity of the remaining galvinoxyl radical signal) for O2 oil, linolenic acid and with and without addition of 0.04 mg of AT and pure α-tocopherol versus time

Surprisingly the O2 sample (refined oil) turned out to be the most efficient radical scavenger (Fig. 1) among the rapeseed oils, although the difference between the scavenging abilities of O2 and other rapeseed oils was not very significant. Such a result was unexpected taking into account the fact that cold-pressed oils (O1 and O3) should present better radical scavenging properties, due to the expected higher antioxidant content than the refined O2 sample. It suggests that O2 contains either more antioxidants than the rest of the oil samples or other reactive systems responsible for galvinoxyl scavenging. Hence, a pure linolenic (LIN3) acid was used by us as a reference standard to estimate the ability of unsaturated fatty acid to quench the galvinoxyl radical (Fig. 2). This process, although appearing to be not very efficient, was associated only with the activity of unsaturated bonds [1, 24]. Nevertheless, the expected higher degree of unsaturated bonds in O2 components (in comparison with O1 and O3) was not confirmed by our further studies (presented below) on the correlation times of the PBN/·lipid radicals as a function of the oil radical structure. Therefore, the galvinoxyl scavenging ability of O2 could be dominated by the presence of phenolic type antioxidants, which is in agreement with the observation that the content of the phenolic antioxidants is much more efficient than the degree of the oil unsaturation [25]. It was additionally supported by us by a distinct acceleration of the galvinoxyl radical quenching upon addition of AT to the oils and fatty acids (Fig. 2). The higher reactivity of the O2 sample suggests a higher α-tocopherol content than that for other rapeseed oil samples.

Furthermore, it appeared that galvinoxyl quenching might be accelerated by other reactive species as was indicated by the more effective radical scavenging ability of the mixture composed of AT and linolenic acid (LIN3-AT) than that for pure AT (Fig. 2).

### Spin Trapping in Oils Solutions

**Fig. 3:**
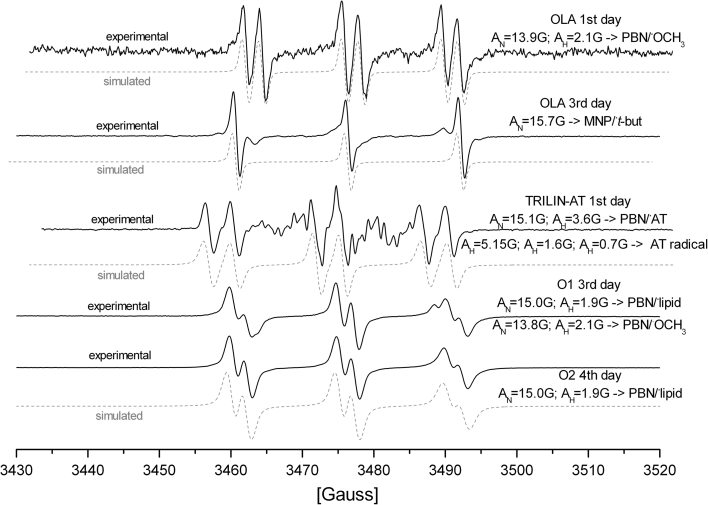
Experimental (*line*) and simulated (*dash*) EPR spectra of different PBN adducts formed during oxidation of oils, triglyceride and fatty acid (hexane solution). For TLIN-AT apart from data for PBN adducts hyperfine constants (hydrogens coupling) for the AT radical are given

The PBN adducts with the radicals generated in oil solutions exhibited distinct variation of the isotropic hyperfine interaction parameters (Fig. 3). It suggests that different radicals were formed and trapped during the oxidation of the oil samples. Three main types of the PBN adducts were detected in the samples containing H_2_O_2_, oils, solvents and α-tocopherol (Fig. 3). One of them was identified as the adduct of pure solvent radical from acetone (AC) or ethyl acetate (EA). The EPR parameters, A_N_ = 13.5 G; A_H_ = 2.1 G for EA and A_N_ = 13.8 G; A_H_ = 2.3 G for AC, are typical for oxygen-centered [26] radicals of not expanded structure [27]. The parameters were assigned previously by us [16] to PBN/·OCH_3_ adduct. Small differences in the isotropic hyperfine parameters values are caused by the solvent effect (smaller E_T_(30) for EA) [20] characterized more detailed below. Interestingly, the value of the *g* parameter for these adducts (2.00615) is even higher than for more expanded structure such as PBN adducts with lipid or α-tocopherol origin radicals (with *g* parameters of about 2.0059). The PBN/·OCH_3_ adduct was not EPR detected in ethanol which most likely plays a role of scavenger of the radicals from H_2_O_2_ [28]. The PBN/·OCH_3_ adducts were not observed the EPR spectra neither in acetone nor ethyl acetate after addition of α-tocopherol (in AC-AT and EA-AT systems, respectively) indicating its quenching activity towards ·OCH_3_ radicals. It is noteworthy that only in AC-AT but not in EA-AT, the second radical adduct PBN/·AT was observed in the spectrum (A_N_ = 15.1 G; A_H_ = 3.6 G). These phenomena could be explained by competition between the reaction of radicals (·OH and ·OCH_3_) with AT and creation of PBN adducts or subsequent reactions between these radicals in the mixture. In EA, a greater number of ·OCH_3_ radicals was formed and the ·AT radicals would be scavenged completely.

The analysis of the EPR spectra of oils solutions also revealed the formation of the PBN adducts with lipid radicals. However, despite the strong oxidation effect of ·OH radicals (as a result of a high concentration of H_2_O_2_) [29, 30] the lipid adducts were recorded not earlier than on the 3rd or 4th day after initiation of the reaction. Before that, only the PBN adducts with ·OCH_3_ and **·**AT radicals from solvents and α-tocopherol, respectively, were detected. The spectral parameters of PBN/·lipid adducts, given in Table 1, are typical for the radicals of long chain compounds (smaller A_H_) with the higher electronegativity of substituents due to relatively greater A_N_ [27]. The parameters remain in good agreement with those reported previously for similar radicals [1, 26], however, small, but noticeable differences should be assigned to the dissimilarity of the solvents; e.g. in ethyl acetate the values of A_N_ and A_H_ were lower than those observed in ethyl alcohol, a more polar solvent with higher E_T_(30) [31]. The solvent effect revealed herein is in good agreement with works described by Janzen, discussing other than lipid types of radicals (phenyl, diphenylmethyl *tert*-butyl aminoxyl, etc.) trapped by PBN [19, 20, 32].

**Table 1:** Experimental isotropic hyperfine coupling constants (in Gauss) of PBN adducts for radicals generated from different oils dissolved in ethyl acetate (EA), acetone (AC) and ethyl alcohol (AL). Attached polarity parameters E_T_(30) [28]

	A_N_ (G)			A_H_ (G)		
	EA	AC	AL	EA	AC	AL
MW	14.9	15.1	15.3	2.0	1.8	2.0
O1	14.0	15.0	15.2	2.0	1.9	2.2
O2	14.4	15.0	15.2	2.0	1.9	2.2
O4	14.4	15.0	15.2	1.8	1.9	2.1
E_T_(30)*[kcal/mol]	38.1	42.2	51.9			

Although the adducts with lipid radicals observed for different oils had similar hyperfine parameters, small variations in the time of their appearance were found. The adducts were formed as the earliest (even on the 2nd day) in the sample of the waste oil (W) where the radical scavenging measurements proved the smallest antioxidant content. Also for the same sample, the intensity of the EPR adduct signals was the smallest and the signals disappeared as the first as compared with those observed for the remaining oils. The rapeseed oils, independently on the production process, exhibited PBN/·lipid adducts a day or two later than the waste oil, but the intensity of the signals was about twice as high. The strongest EPR signals were observed for the solutions of O1. In acetone solutions some of the spectra both adducts: PBN/·OCH_3_ (from acetone solvent) and PBN/·lipid were recorded at the same time. The simulation of the spectra allowed us to determine the hyperfine splitting parameters of the overlapping signals but accurate determination of concentration of the adducts (for e.g. O1, 3rd day in Fig. 3) is saddled with inaccuracy.

### Standard Fatty Acids and Triglycerides Oxidation

The EPR spectra of PBN adducts with the radicals generated by H_2_O_2_ in LIN3, LIN2, OLA, TRILIN, TRIOLA and in their LIN3-AT, OLA-AT, TRILIN-AT, TRIOLA-AT systems (after addition of α-tocopherol) were similar to those observed in the waste and crude oils. For AC and EA solutions in the beginning of the oxidation process, the radicals originated from solvent and later from the lipids were trapped by PBN.

The biggest resistance to oxidation was shown by OLA samples. Apart from the solvent PBN/·OCH_3_ adduct, a very weak signals of the PBN/·lipid adduct were observed, but only in EA. In consequence, the HFCC parameters were determined with little accuracy (A_N_ = 15.1–14.5 G; A_H_ = 1.9–3.0 G). Due to this fact, it was impossible to follow the solvent effect for OLA samples.

The sensibility of the fatty acids and triglycerides to oxidation was evidently dependent on their structure. It was the highest for linolenic acid LIN3 with three unsaturated bonds and for TRILIN with two unsaturated bonds in each fatty acid chain, and the smallest for OLA (with one unsaturated bond) as well in TRIOLA (with one unsaturated bond in each fatty acid chain). The adducts were recorded on the 2nd day for LIN3, whereas the trace signal was observed for LIN2 and no (or very weak) signal for OLA. Additionally, the HFCC parameters were apparently dependent on the unsaturation of fatty acid chain, especially A_N_ value increased in the order 14.70, 14.86 and 14.89 G (in EA as solvent) for OLA, LIN2, LIN3, respectively. Although the changes of A_N_ parameter were not tremendous, they were substantial enough to suggest an increase in spin density of the unpaired electron on the nitrogen atom. Thus, the most unsaturated acid LIN3 revealed the highest A_N_ value indicating a higher spin density on the nitrogen atom than for other more saturated acids. This effect is probably observed as the result of the creation of a more stable polar form in that according to Janzen [27], the value of A_N_ depends on the electronegativity of the attached group.

Similarly, the adducts' spectra for TRIOLA were observed a few days later than those for TRILIN. The HFCC parameters of the PBN/·lipid adducts were similar for TRILIN and TRIOLA (A_N_ = 15.2–14.9 G; A_H_ = 1.8–2.2 G, respectively) and were in good agreement with those for the oils (Table 1). The signal intensity of the adduct formed in triglycerides solutions was always at least ten times higher than for the respective adducts in fatty acids. The signal intensity for TRIOLA adducts was even 100 times higher than for the OLA. The study of fatty acids and triglycerides oxidation was used for standardization of the process and was very helpful in the interpretation of the results obtained for the oils. The adducts of W oil revealed the smallest intensity of the EPR signals and the durability due to its more saturated structure and high free fatty acids content. Rapeseed oils consist mostly of triglycerides thus the intensities of their adduct signals were higher. Hence, the effect of their structural characteristic associated with unsaturated bonds prevails over their adducts' stability.

### Effect of α-Tocopherol Addition on Spin Trapping

**Fig. 4:**
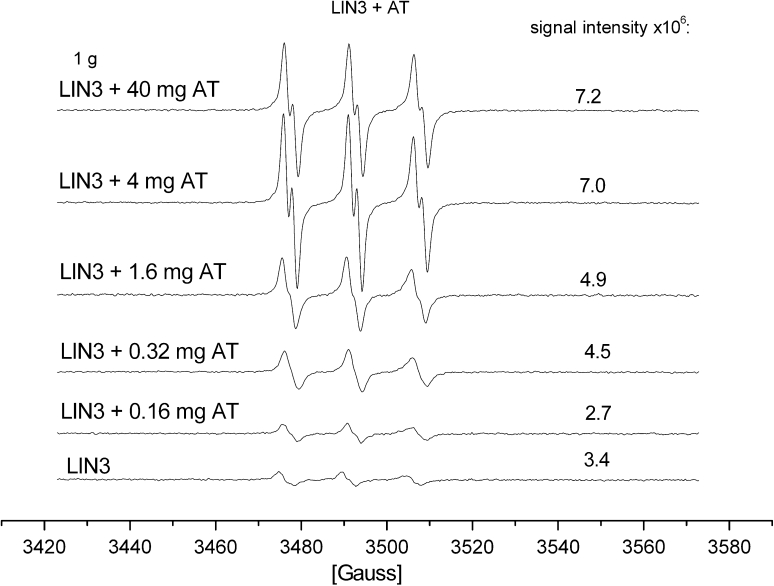
EPR spectra of linolenic acid (LIN3) in acetone with different amounts of α-tocopherol added (AT)

The interesting results were observed on the addition of different amounts of α-tocopherol (0.16–40.0 mg) per 1 g of the oils, fatty acid and triglycerides. Although, the same lipid radicals were trapped by PBN, a huge differentiation in the signal intensities was found (Fig. 4). An addition of the small α–tocopherol quantities (0.16–0.352 mg/1 g of the substance) inhibited the oxidation process and, in consequence, much weaker EPR signals were observed than in the case of a lack of AT (Fig. 4) after the same period of time. The PBN spectrum appeared day or two later than in the case of the sample without α-tocopherol. For the oil samples, the intensity of the signals for PBN/·lipid adduct was about ten times greater than for that upon the addition of a small amount of AT to the same samples. For the resulting OLA-AT, the EPR detection of the radical adducts with PBN was difficult, since the adducts' spectral signals were already weak for OLA. But the signal intensity increased when higher quantities of AT were added. This indicates that the prooxidative effect increased with an increasing amount of α-tocopherol. Hence, under the same conditions (of solvent effect and the studied substance concentration) a differentiation between a positive and negative effect of AT on the oxidation was observed between 0.16 and 0.8 mg per 1 g of the fatty acid or the triglyceride. The mixtures with low AT content (below 0.16/1 g) created the lipid adduct 2–4 days later than the samples with a higher AT content. The EPR signals intensities were the highest for the greatest AT content, while the lowest intensity (lower than for the sample without AT addition) was for samples with low AT content.

The OLA sample, apart from a very weak intensity of the lipid adducts signals, also exhibited the EPR spectrum of another hyperfine splitting pattern (Fig. 3, OLA 3rd day). The spectrum consists of three lines without hydrogen hyperfine splitting which is typical for the MNP/·*t*-but radical adduct. It may indicate that PBN was oxidized in the OLA solutions and MNP spin trap was formed [16], suggesting also that competition between fatty acid and PBN molecule for reaction with ·OH radical occurred. The addition of α-tocopherol in small quantities did not influence this effect. When an excess of **AT** was used (10/250 mg) this competition between fatty acid and PBN oxidation was shifted in support of fatty acids and formation of PBN/·lipid adducts dominating the EPR spectra was observed (as described above).

### Rotational Correlation Time

**Fig. 5:**
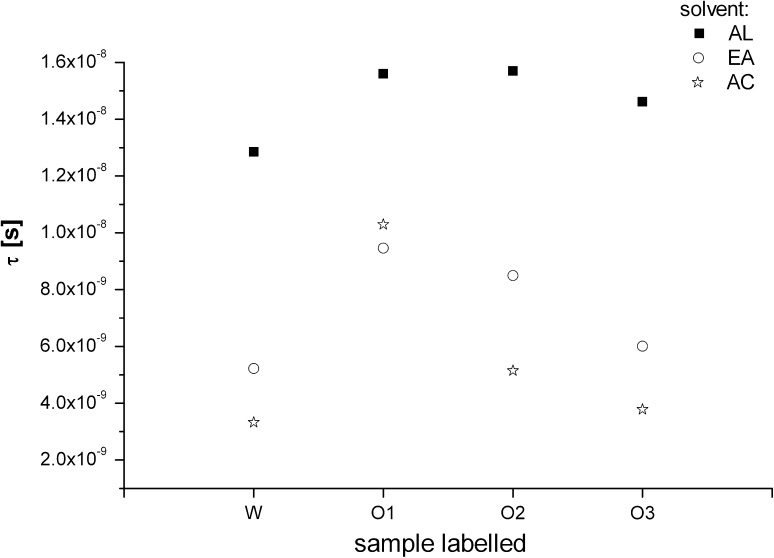
Comparison of the rotational times (τ [s]) calculated from the EPR spectra for PBN adducts with the radicals generated in different oil samples as a function of the solvents

The correlation rotational time τ (s) of the PBN/·lipid adducts, calculated on the basis of the parameters characterizing the shape of ^14^N hyperfine splitting lines of the EPR spectra revealed additional difference between the oil samples. Discussed below, the rotational times are the average result of the parameters calculated for the five-times replicated spectra. Regardless of the oils sample dilution, the resulting PBN radical adducts exhibited different *τ* depending on the sample composition, indicating a different structure of the trapped lipid radicals. The smallest value of this spin-labeling parameter was observed for the W sample indicating its smallest molecular viscosity (Fig. 5). Surprisingly, very similar result were found for the O3 sample. The *τ* values were significantly dependent on the solvent (the highest *τ* values were observed for solutions of the highest solvent polarity), but the tendency stayed the same for the particular oil sample. The highest *τ* values were found for the O1 oil. It indicates that in this mixture, the motion of the PBN adduct molecules was the most efficiently inhibited. The *τ* values for the standard fatty acids and triglycerides solutions appeared to be distinctly dependent on their chain expansion and saturation.

In general, the highest *τ* (the slowest molecule motion) was observed for the triglycerides samples, whereas the fatty acids created more flexible adducts. On the other hand, the more stable (existing for longer time) PBN adducts with the smaller *τ* values were formed by the fatty acids and their triglycerides with the higher unsaturated bonds content, like LIN3 (*τ* = 1.9 × 10^−9 ^s) and TRILIN (*τ* = 3.9 × 10^−9 ^s). In these samples the adduct rotation was much more efficient, resulting in even ten times smaller *τ* values than those for OLA (*τ* = 2.0 × 10^−8 ^s) and TRIOLA (*τ* = 1.1 × 10^−8 ^s).

Inhibition of PBN/·lipid molecular motion for OLA and TRIOLA correlates with a delay in the adduct formation and a lower EPR signal intensity of the adducts.

The unsaturated bond, responsible for the oxidation reaction, is in OLA situated in the middle of the acid chain. The lipid radical formed easily as the first one due to the small size of the attacking reactive oxygen species could then encounter steric hindrance during the reaction with PBN. The created adduct was then unstable and easily underwent the recombination giving a weak EPR spectrum which disappeared quickly. A totally different situation appears in a LIN3 sample where the polyunsaturated structure of linolenic acid causes its higher reactivity. Additionally, the unsaturated bonds in LIN3 are situated on one side of the molecule, thus a steric hindrance does not exist and the created lipid radical is more easily caught by the spin trap and exists for a longer time.

Comparing the EPR characteristics of the PBN adducts with those for waste and rapeseed oils it is noteworthy that the smallest *τ* time and instability of the EPR signal indicate a high content of the free and saturated acids in W, while, in the O1 sample, the highest correlation time and longer stability of the signal proves the presence of a higher degree of saturated components but in combination with a more expanded triglycerides structure. The oil sample O2 exhibited a *τ* value close to that for O3 and similar to the unsaturated triglycerides such as TRILIN.

## Conclusions

The processes of the oxidation of the oil samples followed by the oxidation of their components (fatty acids and triglycerides) were investigated by advanced EPR techniques such as spin scavenging, trapping and labeling. The EPR signal intensities, hyperfine splitting parameters and correlation rotational times of the PBN radical adducts were used for identification of the radicals formed during oxidation of the edible oils. The HFCC parameters derived by simulation of the experimental spectra allowed us to differentiate the fatty acids depending on the degree of their unsaturation. The acids with higher numbers of unsaturated bonds exhibit higher A_N_ values of the PBN/·lipid adduct.

The scavenging activity of the oils towards galvinoxyl radicals being the measure of their antioxidant properties appeared to be independent of the method of oil production. The concentrations of α-tocopherol naturally occurring in oils were appropriate to exhibit a positive antioxidant effect.

The EPR studies of oxidation of fatty acids or triglycerides with α-tocopherol (AT) by the radicals generated from H_2_O_2_ proved that this process is inhibited by small concentrations of AT, whereas it is accelerated by higher concentrations of AT (greater than 0.16 mg AT/1 g). Additionally, the EPR spectra of the PBN adducts allowed us to differentiate the properties of lipid radicals depending on the parent fatty acids' unsaturation. The oils with a higher amount of unsaturated acids created more stable adducts. The oils with a high content of free fatty acids, like waste oils, underwent the oxidation reaction much more easily, but due to a high content of saturated acids, the radicals formed were not stable and were detected by EPR for shorter periods of time than those generated in crude vegetable oils.
